# Supplementary material for: Tunable Fluorescence-Responsive Double Hydrophilic Block Polymers Induced by the Formation of Pseudopolyrotaxanes with Cucurbit[7]Uril
Source: Polymers (Basel). 2019 Sep 9;11(9):1470. doi: 10.3390/polym11091470 (PMC6780542; doi:10.3390/polym11091470)
Supplement: Supplementary file 1 [file polymers-11-01470-s001.zip › Supplementary Material.docx]

**Supporting information for**

**Tunable fluorescence responsive of double hydrophilic block polymers induced by the formation of pseudopolyrotaxanes with cucurbit[7]uril**

Xiumin Qiu^1^, Xin Wang^2^, Shengzhen Hou^1^, Jin Zhang^1^, Jing Zhou^1^, Yebang Tan^1*^

*^1^ School of Chemistry and Chemical Engineering, Key Laboratory of Special Functional Aggregated Materials, Key Laboratory of Colloid and Interface Chemistry, Ministry Education, Shandong University, Jinan 250100, People’s Republic of China*

*^2^ School of Light Industry and Engineering, Qilu University of Technology, Jinan 250353, People’s Republic of China*

* Address Correspondence to E-mail: [ybtan@sdu.edu.cn](mailto:ybtan@sdu.edu.cn) Tel: +86 531 88363502

1. **Syntheses**

**1.1** The syntheses routes of mPEG-Br (a) mPEG-*b*-P4VP (b) and mPEG-*b*-QP4VP (c) is shown in Scheme S1.

**

**

**Scheme S1**.The syntheses routes of mPEG-Br (a) mPEG-*b*-P4VP (b) and mPEG-*b*-QP4VP (c)

- 1. **Preparation of side-chain polypseudorotaxanes**

The cucurbit[7]uril side-chain polypseudorotaxanes with various composition and concentration is prepared by mixing the pristine copolymer stock solution with quantitative CB[7] suspension and pure water. The synthesis of side-chain polypseudorotaxanes 0.5 mg/mL (with H:G =1:1) solution was used as an example. In order to achive the desired concentration of 0.5 mg/mL, 9.5 mL pure water was added firstly followed by 5 mg mPEG-*b*-QP4VP was dispersed in pure water (10 mL), and 0.5 mL (34 mg/mL) suspension was added. The mixture was mixed with vortex (2500 rpm, 30s).

1. **Characterizations of the polymers**

The ^1^H NMR spectrum of mPEG_43_-Br is shown in Figure S1. From the area of peak e and a as shown in the ^1^H NMR spectrum of Figure S1, it can be concluded that all mPEG_43_-OH has been translated into mPEG_43_-Br.
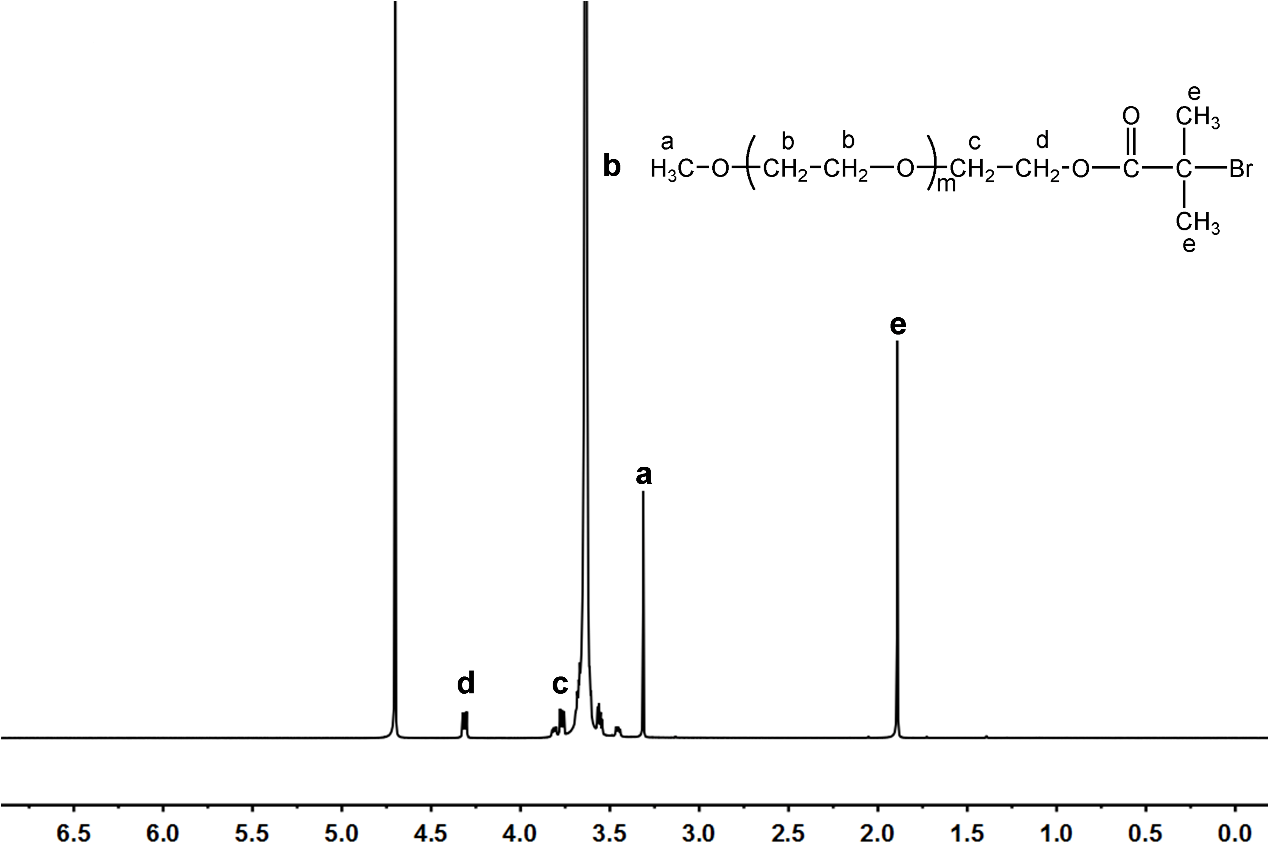


**Figure S1**. ^1^H NMR spectrum of mPEG_43_-Br (400 MHz, CDCl_3_).

The ^1^H NMR spectrum of mPEG_43_-*b*-P4VP is shown in Figure S2. ^1^H NMR (400 MHz, CDCl_3_), δ(TMS, ppm): 8.43 (H_f_, 2H), 6.38 (H_e_, 2H ), 3.64 (H_b,_ 4H), 3.32 (H_a_, 3H), 2.5 (H_d_, 1H), 1.5 (H_c_, 2H). The GPC trace for mPEG-*b*-P4VP in DMF is shown in Fig S3.

**
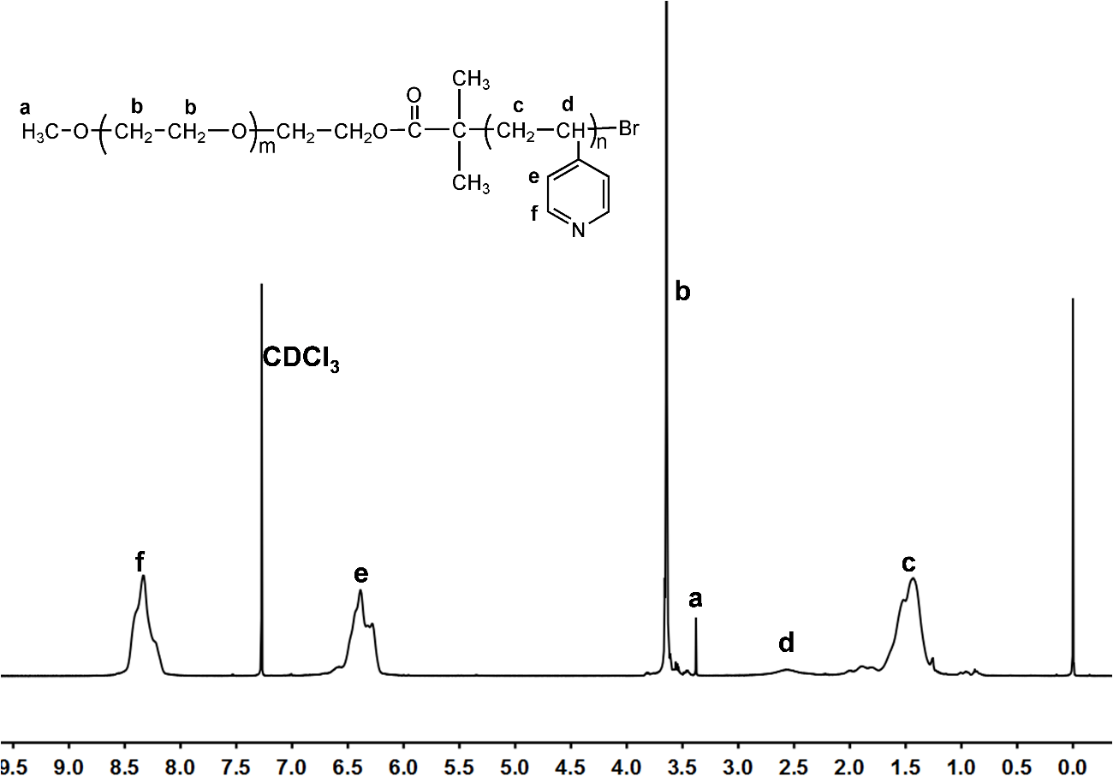
**

**Figure S2**. ^1^H NMR spectrum of mPEG_43_-*b*-P4VP (400 MHz, CDCl_3_).





**Figure S3**. GPC trace obtained for the mPEG-*b*-P4VP.

The ^1^H NMR spectrum of mPEG_43_-*b*-QP4VP is shown in Figure S4. ^1^H NMR (400 MHz, DO_2_), δ (TMS, ppm): 8.57 (H_a’_, 2H), 7.42 (H_b’_, 2H ), 7.78 (H_e_, 2H), 7.39 (H_d_, 2H), 8.44 (H_b_, 2H), 6.51 (H_a_, 2H), 3.64 (H_g,_ 4H), 3.32 (H_h_, 3H), 1.5 (H_f_, 3H).


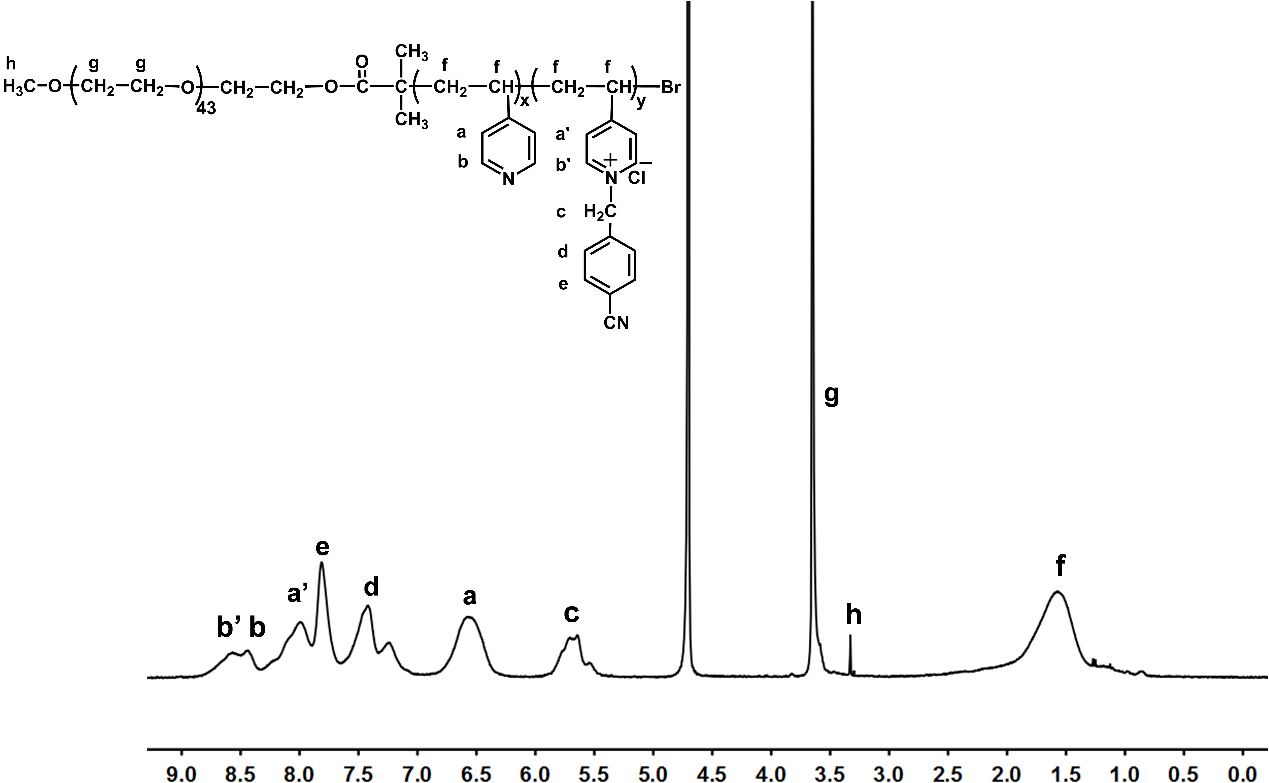


**Figure S4**. ^1^H NMR spectrum of mPEG_43_-*b*-QP4VP (400 Hz, D_2_O).

The FT-IR spectra of mPEG, mPEG-Br, mPEG-*b*-P4VP, quaternized copolymer mPEG-*b*-QP4VP was shown in Figure S5. In the FT-IR spectrum (Figure S5b), the absorption peaks at 3433 cm^-1^ disappeared, and new peaks could be seen at 1732 cm^-1^ (C=O) correspongding to the esters. The result indicated the successfull synthesis of the macroinitiator mPEG-Br. The characteristic absorption peaks at 1600 cm^-1^ and 1559 cm^-1^ (C=N) were observed, suggesting that the polymer (mPEG-*b*-P4VP) was successfully synthesized (Figure S5c). Meanwhile, as shown in Figure S5d, other than the three absorption bands at 1732 cm^-1^ (C=O), 1600 cm^-1^ (C=N) and 1559 cm^-1^, the characteristic absorption of the quaternary N atom was observed at 1638 cm^-1^, which illustrated the successful synthesis of the desired block copolymers.


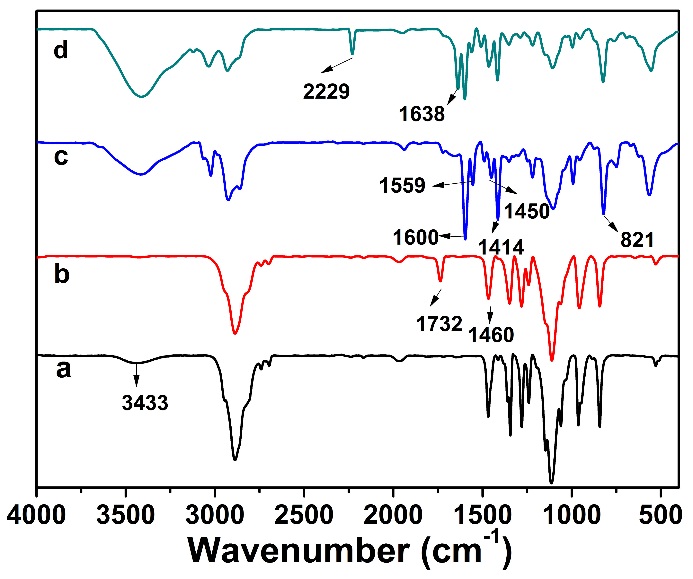


**Figure S5**. FT-IR spectra of mPEG-OH (a), mPEG-Br (b), copolymer mPEG-*b*-P4VP (c), quaternized copolymer mPEG-*b*-QP4VP (d).

The concentration-dependent fluorescence emission spectra of mPEG-*b*-QP4VP in water are shown in Figure S6.
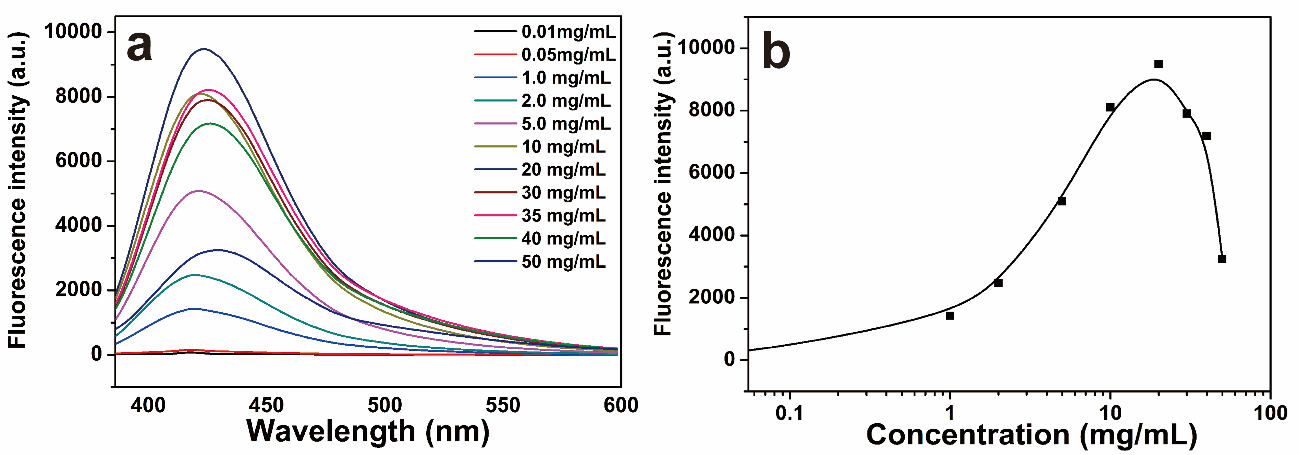


**Figure S6**. (a) Concentration dependent fluorescence emission spectra of mPEG-*b*-QP4VP in aqueous solution at pH 6.0 with excitation at 365 nm. (b) Variation in fluorescence intensity of fluorescence spectra of mPEG-*b*-QP4VP solution as a function of polymer concentrations at 25 °C.

pH response of the mPEG-*b*-QP4VP micelles

As an amphiphilic copolymer, mPEG-*b*-QP4VP can respond to external pH conditions owing to the pyridine groups on the 4-vinyl pyridine (4-VP) segment [[1](#_ENREF_1)]. Low pH (pH < 5.0)-induced protonation of the pyridine ring was able to initiate a hydrophobic to hydrophilic transition, and has been employed widely to construct pH-responsive nanostructures. It was suggested that P4VP has a pK_a_ value of approximately 4–5, rendering it insoluble in neutral and alkaline aqueous media [[2](#_ENREF_2),[3](#_ENREF_3)]. At pH values above the pK_a_, the P4VP chains become hydrophobic and shrink. When the pH decreases below the pK_a_, the polymer chains turn to hydrophilic and stretch owing to the protonation of the 4VP units. Therefore, for the mPEG-*b*-QP4VP copolymer, lower pH values are preferred to trigger possible chemical changes. Therefore, three pH conditions (pH 4.0, 5.0, and 7.4) were chosen to test the pH-responsive abilities of the assembly. As shown in Figure S7b, the pH-responsive fluorescence emission spectra of mPEG-*b*-QP4VP in different buffer solutions were acquired under an excitation wavelength of 365 nm, and one emission peak at 432 nm was recorded. At pH 7.4, weak intensities of the peak resulted from the proximity of the encapsulated 4-cyanobenzyl group in the micellar core, which is likely to cause fluorescence quenching by energy transfer [[4-7](#_ENREF_4)]. When the pH was decrease to 5.0 and 4.0, the hydrophobic P4VP gradually became hydrophilic, leading to partial or complete disassembly of the micelles, which generated stronger intensities of emission spectra. The results illustrated that a simple and effective modification of the free copolymers with fluorescent dyes conferred fluorescent properties to the system. In addition, the fluorescent intensity change was governed by the pH response of mPEG-*b*-QP4VP, further confirming the pH response of the assembly.The hydrodynamic size distribution of mPEG-*b*-QP4VP at pH 4.0, 5.0, and 7.4 is shown in Figure S7c. At pH 7.4, the assembled formulation was stable, and the hydrodynamic size was found to be 146.5 nm, with a narrow PDI of 0.341. The assembly of mPEG-*b*-QP4VP at this pH condition was imaged by TEM, as shown in Figure S7d. At a lower pH of 5.0, the hydrodynamic size was increase to 156.7 nm with a broad distribution (PDI = 0.502, possibly due to the electrostatic repulsion induced by the partially protonated pyridine groups, leading to a large hydrodynamic size and incomplete destruction of mPEG-*b*-QP4VP, as shown in Figure S7c. when the pH was decreased to 4.0, the hydrodynamic size markedly decreased, and the maximum intensity was centred at 11.45 nm, indicating that the hydrophobic-hydrophilic transition of P4VP induced the disassembly of mPEG-*b*-QP4VP. The degraded mPEG-*b*-QP4VP at this pH condition was imaged by TEM, as shown in Figure S7d. The pH-dependent stability of the assembled formulation enabled their use as intelligent carriers for therapeutic agents.


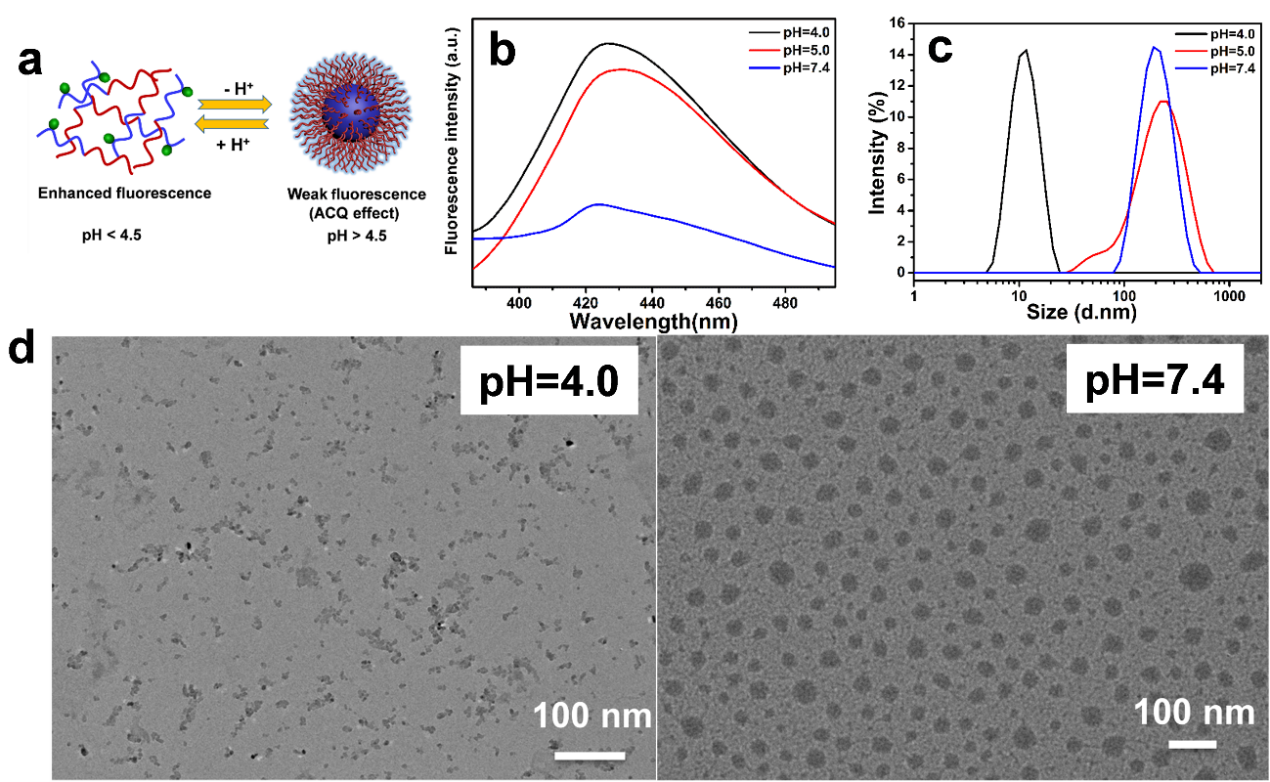


**Figure S7** (a) Schematic illustration of pH-responsiveness of micellar mPEG-*b*-QP4VP. (b) Fluorescence spectra of mPEG-*b*-QP4VP in buffer solutions with different pH values at room temperature; excited wavelength = 365 nm. (c) Hydrodynamic size distribution of the assembly mPEG-*b*-QP4VP at pH7.4, pH 5.0, and pH 4.0. (d) TEM images of the degraded mPEG-*b*-QP4VP in pH 4.0 and assembled mPEG-*b*-QP4VP in pH 7.4 buffers at a polymer concentration 0.5 mg/mL.

3. Binding behavior of mPEG-*b*-QP4VP with CB[7]

The UV-vis absorption spectral changes in mPEG-*b*-QP4VP as a function of added CB[7] are shown in Figure S8.





**Figure S8**. UV-vis spectra of mPEG-*b*-QP4VP and pseudopolyrotaxanes with different content of CB[7] thread.

The decay lifetime of the mPEG-*b*-QP4VP and pseudopolyrotaxanes are shown in Figure S9.





**Figure S9**. Fluorescence decay of mPEG-*b*-QP4VP and mPEG-*b*-QP4VP/CB[7](1:1) in water at λ_ex_ = 365 nm.

4. In vitro cell viability assay

The potential toxicity of mPEG-*b*-QP4VP and pseudopolyrotaxanes were evaluated by using an MTT assay to NIH3T3 cells. As shown in Figure S10, the viability of NIH3T3 and HeLa cells treated with pseudopolyrotaxane micelles for 48 h was over 85% in all tests, even at applied concentrations up to 500 μg/mL. However, the viability of NIH3T3 cells treated with mPEG-*b*-QP4VP for 48 h was lower than 50% only at concentrations up to 200 μg/mL (Figure S10). The results demonstrated that the pseudopolyrotaxane micelles were nontoxic and could be safely used as biocompatible carriers for drug delivery.





**Figure S10**. Viability of NIH3T3 cells incubated with different concentrations of mPEG-*b*-QP4VP and pseudopolyrotaxanes for 48 h.

**5. Drug-loaded micelles**

The morphology and particle size distribution of the blank and drug-loaded micelles were observed by DLS and TEM as shown in Figure S11.


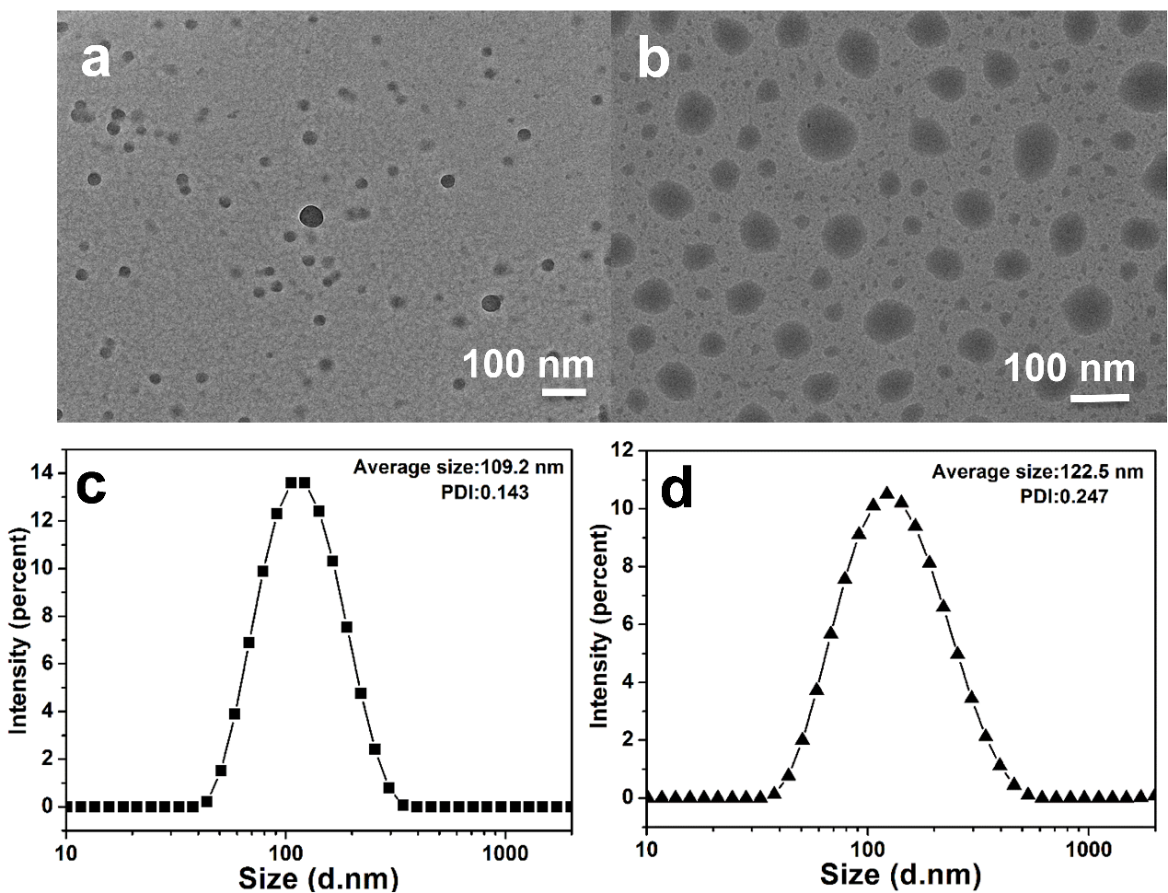


**Figure S11**. (a) TEM image of blank pseudopolyrotaxanes micelles (b) TEM image of DOX-loaded pseudopolyrotaxanes micelles. (c) The size distribution of pseudopolyrotaxanes micelles before and (d) after DOX loading measured by DLS.

**References**

1. Gohy, J.-F.; Antoun, S.; Jérôme, R. pH-Dependent Micellization of Poly(2-vinylpyridine)-block-poly((dimethylamino)ethyl methacrylate) Diblock Copolymers. *Macromolecules* **2001**, *34*, 7435-7440, doi:10.1021/ma010535s.

2. Sidorov, S.N.; Bronstein, L.M.; Kabachii, Y.A.; Valetsky, P.M.; Soo, P.L.; Maysinger, D.; Eisenberg, A. Influence of Metalation on the Morphologies of Poly(ethylene oxide)-block-poly(4-vinylpyridine) Block Copolymer Micelles. *Langmuir* **2004**, *20*, 3543-3550, doi:10.1021/la0360658.

3. Zhao, L.; Li, A.; Xiang, R.; Shen, L.; Shi, L. Interaction of FeIII-Tetra-(4-sulfonatophenyl)-porphyrin with Copolymers and Aggregation in Complex Micelles. *Langmuir* **2013**, *29*, 8936-8943, doi:10.1021/la401805x.

4. Zhan, X.; Yi, Q.; Cai, S.; Zhou, X.; Ma, S.; Lan, F.; Gu, Z.; Wu, Y. Polymer-entanglement-driven coassembly of hybrid superparamagnetic nanoparticles: Tunable structures and flexible functionalization. *J. Colloid Interface Sci.* **2017**, *508*, 263-273, doi:https://doi.org/10.1016/j.jcis.2017.07.116.

5. Park, J.-H.; von Maltzahn, G.; Ruoslahti, E.; Bhatia, S.N.; Sailor, M.J. Micellar Hybrid Nanoparticles for Simultaneous Magnetofluorescent Imaging and Drug Delivery. *Angewandte Chemie* **2008**, *120*, 7394-7398, doi:doi:10.1002/ange.200801810.

6. Gao, G.; Heo, H.; Lee, J.; Lee, D. An acidic pH-triggered polymeric micelle for dual-modality MR and optical imaging. *J. Mater. Chem.* **2010**, *20*, 5454-5461, doi:10.1039/C0JM00317D.

7. Zhou, K.; Liu, H.; Zhang, S.; Huang, X.; Wang, Y.; Huang, G.; Sumer, B.D.; Gao, J. Multicolored pH-Tunable and Activatable Fluorescence Nanoplatform Responsive to Physiologic pH Stimuli. *J. Am. Chem. Soc.* **2012**, *134*, 7803-7811, doi:10.1021/ja300176w.
